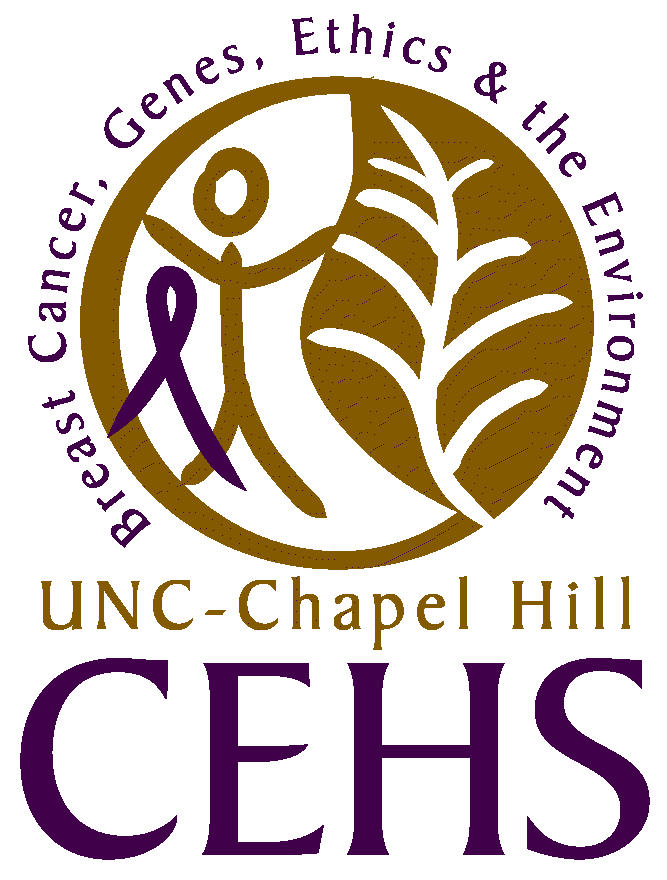# Beyond the Bench: Help Instead of Hype

**DOI:** 10.1289/ehp.112-a992

**Published:** 2004-12

**Authors:** Erin E. Dooley

Breast cancer strikes 1 in 7 American women, making it the most commonly diagnosed cancer among women in the United States. Although certain genetic factors can play a part in the etiology of the disease, scientists are studying other important factors including estrogen-related factors, lifestyle choices, and environmental risk. Understanding these factors can help women make informed decisions about their health and avoid being another breast cancer statistic.

Researchers at the NIEHS-funded Center for Environmental Health and Susceptibility, housed in the University of North Carolina at Chapel Hill School of Public Health, are leading the way in determining both the genetic and other causes of breast cancer. And the center’s Community Outreach and Education Program (COEP), headed by Frances M. Lynn, a professor of environmental sciences and engineering, has developed a workshop program to spread the word to North Carolina residents that they need not be helpless victims of this disease.

To assist in developing the workshop, the COEP partnered with the Breast Cancer Coalition of North Carolina, a nonprofit organization that advocates on behalf of those with breast cancer and their families. A scientific advisory board representing a variety of medical disciplines reviewed the workshop materials and continues to work with COEP staff to answer participants’ questions and keep the workshop as current as possible.

Visitors to the center’s website can download the workshop materials at **http://www.sph.unc.edu/cehs/outreach/elsi.htm**. Visitors can download the 15-slide PowerPoint presentation that is used in the workshop, as well as an agenda, facilitator instructions, case studies, fact sheets, and take-home activities.

The presentation introduces the workshop audience to the known possible risk factors for breast cancer, as well as some risk-reduction measures women can take. The presentation divides risk factors into four groups: personal or estrogen-related risk, lifestyle risk, environmental risk, and genetic or inherited risk.

The environmental risk portion of the presentation explains gene–environment interactions that occur as a result of exposure to toxicants and how that differs from risk associated with inheriting one of the so-called breast cancer genes (*BRCA1* or *BRCA2*). Despite the frightening prospect of breast cancer running in families, only 5–10% of breast cancer cases are thought to be genetic in origin. Slides describe instances where this inherited risk may be implicated in breast cancer. Participants also learn about the ethical, legal, and social implications of genetic testing—how the testing is done, how they should decide if they need it, and what may happen if they test positive.

The interactive portion of the workshop includes fun learning activities, such as Reduce Breast Cancer Bingo, which has been a hit with the senior citizens that have taken part in the program to date. Participants win when they correctly identify four risk-reduction facts in a row, including the importance of exercising, eating vegetables, and limiting exposure to secondhand smoke. A related activity presents participants with fictional case studies for three women, one with a family history of breast cancer, one with lifestyle risk factors, and one with environmental risk factors. Participants are asked to identify both the risk factors and any protective factors each women has, and to recommend how each woman might reduce her risk.

The workshop, which has been conducted across North Carolina, has been developed so that women who have completed it know not only how to better care for themselves but also how to advise other women. In conducting the workshops, COEP staff hope to dispel some of the myths women have about breast cancer and instill optimism instead.

## Figures and Tables

**Figure:**